# Diversity of fishery resources and catch efficiency of fishing gears in Gorai River, Bangladesh

**DOI:** 10.1016/j.heliyon.2021.e08478

**Published:** 2021-11-27

**Authors:** Kishor Kumar Tikadar, Mrityunjoy Kunda, Sabuj Kanti Mazumder

**Affiliations:** a Department of Fishery Resources Conservation and Management, Khulna Agricultural University, Khulna, 9100, Bangladesh; b Department of Aquatic Resource Management, Sylhet Agricultural University, Sylhet, 3100, Bangladesh; c Department of Genetics and Fish Breeding, Bangabandhu Sheikh Mujibur Rahman Agricultural University, Gazipur, 1706, Bangladesh

**Keywords:** Abundance, CPUE, Catch composition, Margalef's richness index, Pielou's evenness index, Shannon-weaver diversity index, Simpson's index

## Abstract

Gorai River is one of the important rivers in Bangladesh for rich aquatic biodiversity. The river is originated from the Ganges-Padma River system, a trans-boundary river between India and Bangladesh. Once the river was rich in fish biodiversity, but due to man-made and natural causes the availability of fish reduced drastically. A comprehensive analysis of fish diversity indices, gear efficiency, catch composition and decline causes of fish diversity in Gorai River, Bangladesh was accomplished. The data were collected on monthly basis from January to December 2018 from three major fishing sites of the river. A total of 62 fish and 2 prawn species under 12 orders and 24 families were recorded. Cypriniformes was the leading order consisting 27% of the total catch. The mean values of Shannon-Weaver diversity (H′), Simpson's index (1-D), Margalef's richness (d) and Pielou's evenness (J′) indices were recorded as, 1.478 ± 0.495, 0.57 ± 0.197, 15.115 ± 4.435 and 0.481 ± 0.152, respectively. At the similarity of 58.7%, two groups were attained in the cluster analysis and the Non-metric Multidimensional Scaling (nMDS) showed 40% similarity among the three sites in twelve months based on the Bray-Curtis similarity matrix. The highest and lowest CPUE were recorded from seine net (5.2 ± 1.72 kg gear^−1^ haul^−1^) and hook & long lines (0.0135 ± 0.0015 kg gear^−1^haul^−1^), respectively whereas, highest and lowest gear efficiency were recorded from lift net (0.321 ± 0.036 kg gear^−1^person^−1^hour^−1^) and fish trap (0.0005 ± 0.0002 kg gear^−1^person^−1^hour^−1^), respectively. Alternatively, the highest fish catch was recorded on April (21228 ± 464.38 kg) and lowest on August (3855 ± 138.21 kg). According to the fishermen fish biodiversity of the Gorai River declined day by day due to overexploitation, destructive fishing practice, pollution, construction of obstacles for fish movement, and natural causes like siltation. Proper implementation of fish acts and regulations, use of authorized fishing gear, community-based fisheries management, sanctuary establishment and management, stocking of fish fingerling, and raising public awareness can play a great role in enhancing and conserving fish biodiversity in the Gorai River of Bangladesh.

## 1 Introduction

Bangladesh, with its large river systems, has significant capture fishery potential and the suitable geographic location of Bangladesh comes with a large number of fish and other aquatic species (Shamsuzzaman et al., 2017). Total fish production of Bangladesh in 2018 was 4.1 million MT, placing it fourth in open water and fifth in aquaculture fish production in the world (Mredul et al., 2020). In the face of the possession of exceptionally productive inland waters of around 45,000 km^2^, the proceeding with a decline in fish catch progressively undermines the livelihoods of over 12 million fishermen in Bangladesh (Hossain et al., 2006). Gorai River is one of the most important rivers in Bangladesh that is originated from the Ganges-Padma River system, a transboundary river between India and Bangladesh. Once the river was rich in fish biodiversity and the livelihood of thousands of fishers' families were fully dependent on Gorai River, but in the recent years due to many man-made and natural causes the availability of fish was drastically reduced (Hanif et al., 2016). But very few researches were conducted on fish biodiversity status of the Gorai River. Therefore, this study has been conducted to find out the history and present status of fish biodiversity along with the causes of loss of biodiversity over the years. The Gorai River ecosystems originated from the Ganges-Padma River system play a vital role in supporting the biodiversity of fish fauna and contribute to the supply of animal protein and overall economy of the south-western part of this country through fish production (Hanif et al., 2016; Rahman et al., 2012). All along with the world, freshwater conditions are encountering genuine dangers of biodiversity and environmental security and numerous techniques have been proposed to tackle this emergency (Suski and Cooke, 2007; Sarkar et al., 2008). In the last few decades, stress caused by anthropogenic degradation due to urbanization, construction of dams, abstraction of water for irrigation and power generation, and pollution had many negative impacts on the biodiversity of freshwater fish species in the river (Sarkar et al., 2008). Recently, the importance of monitoring biodiversity in the protected areas has been realized by the developing countries (Danielsen et al., 2000).

Diversity index provides more information than simply the number of species present in a particular water body which acts as an important tool that gives vital information on the scarcity and commonness of species in a community (Sultana et al., 2018). It is well known that the catch per unit effort (CPUE) is a measure of stock density, physical and financial productivity, and an indicator of the efficiency of a fishing operation (Ghosh and Biswas, 2017). CPUE is a useful index of the abundance and exploitation of fishery resources to determine the number of fishing units of a sustainable fishery. CPUE is expected to be proportionate to the fish population that is utilized as the relative abundance index (Karim et al., 2019). Assessment of species abundance and biomass usually give an outline of the population structure that exists in the water bodies (Saha et al., 2018). There was no study found on CPUE and efficiency of fishing gear used to catch fish in the Gorai River of Bangladesh. But some researches were conducted on the CPUE and gear efficiency in different waterbodies of Bangladesh (Ahmed and Hambrey, 2005; Ahmed, 2008; Galib et al., 2009; Sayeed et al., 2014).

While the loss of biodiversity continues globally (Tittensor et al., 2014), the need for evidence-based decision-making in the environmental sector is increasingly recognized (Lundquist et al., 2015; Perrings et al., 2011). Management of open water fisheries has been a problem confronting all fisheries countries around the world. Fisheries management refers to a bunch of lawful, social, financial, and political game plans for the management of fisheries at local, domestic and international levels (Huang and He, 2019). There is a need for greater emphasis on dialogue and mutual learning between researchers and decision-makers to increase the policy impact and ultimately the societal impact of ecological research. This dialogue must include the entire information generation process through scientific research, policy design, and implementation. There is also a need for better framing of the science-policy interfaces (SPIs) to increase transparency, address potential limitations and procedural biases and assess the progress made in such collaborative undertakings (Carmen et al., 2015; Nesshö ver et al., 2016; Schindler et al., 2016). However, very few efforts have yet been made to recognize the status of fish diversity of Gorai River with potential effects and declining reasons for fish species.

Hence, it is important to do logical work concerning accessible fishing gears including their work measure, catch per unit effort (CPUE), gear efficiencies, catch composition of various angling gear, all-out fish catch, fish diversity index, and some potential effects in charge of decreasing fish fauna to speak to pattern information to shield the fisheries decent variety to close eradication of the river. Considering all the thrust issues, the objectives of the study were to assess fish assemblages, diversity, CPUE, gear efficiencies, catch composition of various gears, the significant reasons for the eradication of fish fauna, and find out some recommendations to enhance the fish biodiversity of the Gorai River in Bangladesh. This study was mainly designed to provide answers to the following questions:

1. Which species of fish and prawns are present in the Gorai River of Bangladesh?
2. Which species are most abundant in the study area?
3. What types of fishing gears and crafts are used to catch fish with their basic features, mode of operations, CPUE, and gear efficiency of fishing gear, effects of fishing gear on fish biodiversity, and total fish catch of the three study sites?
4. What are the main reasons for fish biodiversity depletion in the Gorai River of Bangladesh with their solutions and recommendations?

## 2 Materials and methods

### 2.1 Ethical approval

The Ethics Committee of the Department of Aquatic Resource Management, Sylhet Agricultural University, Sylhet, Bangladesh, approved the specific experimental design.

### 2.2 Study area and data collection

**Figure 1:**
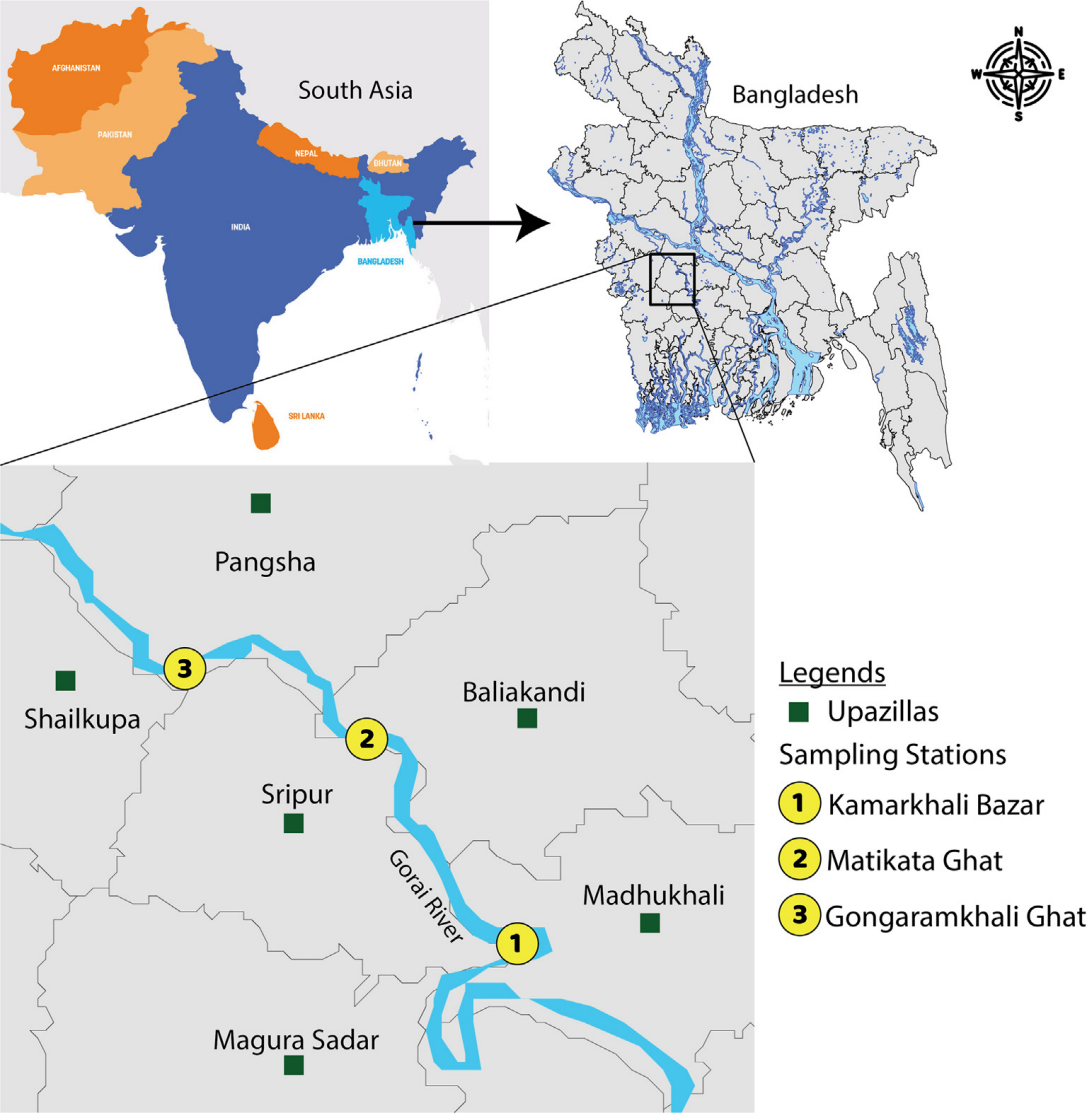
Map showing study area in the Gorai River of Bangladesh.

The study area was including about 50 km of the river area from Gongaramkhali Ghat of Magura district to Kamarkhali Bazar of Rajbari District. Three sampling stations named Site 1- Kamarkhali Bazar (23°32′ N, 89°33′ E), Site 2- Matikata Ghat (23°35′ N, 89°30′ E) and Site 3- Gongaramkhali Ghat (23°35′ N, 89°29’ E) were selected for the study (Figure 1). The study was conducted for a period of twelve months from January 2018 to December 2018. The study was guided by the data collected from the direct catch assessment survey (CAS) and fishing effort survey (FES) conducted at the study sites. The fishing gears were overviewed by direct physical perceptions and dependent on participatory rural appraisal (PRA), for example, questionnaire interview (QI), focus group discussion (FGD), and cross-checking of data by key informant interviews (KII). During the study period, a total of 80 fishermen, 25 Aratders and 40 fish traders were randomly selected for questionnaire interviews from 7 villages and 5 fish market surrounding the study area. The questionnaire interviews were done at household, during fishing in the river, and market places depending on the presence of the fishermen and fish traders. Focus group discussion (FGD) refers to objective focusing informal discussions with small groups (6–12) of people, generally with same vocation or belonging to same level of community. A total of seven (7) FGD were made from 7 villages of the study area. Each of the FGD performed with 6–12 members. The participants of the FGD were fisherman of young, middle and old ages. The QIs and FGDs were led with a semi-organized and pre-trialed questionnaire.

### 2.3 Catch assessment and fishing gear survey

Catch assessment information was collected by direct observation of fish species caught by the fishermen using different types of fishing gear like seine net, drifting gill net, gill net, lift net, fish trap, hook, and line, and wounding gear also. Data were collected only from small-scale and artisanal fishermen but not from any fishing industries. Fishing Effort Survey (FES) and Catch Assessment Survey (CAS) were carried out by using a vessel from 7 am to 5 pm twice a month throughout the year. Every inspection was done in a similar locality with three replicates. The number of fishermen catches recorded was not the same in each month because their number was varying with the types of fishing gear were used to catch fish. Different types of fishing gear used in different months or seasons to catch fish. Samples were gathered entirely for little catch and various sub-sample for huge catch legitimately from fishers during fishing. Weight of fish was measured by using a digital balance. All out weight of catch, time of fishing, the span of fishing, individuals connected with each gear, number of species caught, number of individuals of each species per unit weight, number of fishing efforts of each gear were recorded. In case of little catch, the total catch was arranged by the number and weight of every species. A huge catch was surveyed by taking at least one sub-sample.

### 2.4 Fish abundance and biodiversity status

In this study, the Shannon-Weaver diversity index (H′), Simpson's index (1-D), Pielou's evenness index (J′) and Margalef's richness index (d) were calculated on monthly sampling in each site for understanding the status of fish diversity using the following formulas:

Shannon-Weaver diversity index, H' = -∑ Pi ln Pi (Shannon and Weaver, 1949)

Where, H’ is the diversity index and Pi is the relative abundance (s/N), where, s is the number of individuals of one species and N is the total number of individuals in the sample. The Shannon-Weaver diversity index is one widely used index for comparing diversity between various habitats (Clarke et al., 2014). It assumes that individuals were randomly sampled from an independent large population, and all the species were represented in the sample (Shannon and Weaver, 1949).

Simpson's dominance index is often used to quantify the biodiversity of habitat which takes into account the number of species, as well as the abundance of each species (Vijaylaxmi et al., 2010). The formula used for calculating is:

Simpson's index, 1-D = 1–(∑ *n* (*n*–1) / *N* (*N*–1)) (Simpson, 1949)

Where, n = the total number of organisms of a particular species, N = the total number of organisms of all species.

Margalef's richness index, d = S-1/ln N (Margalef, 1968)

Here, d is the richness index, S is the total number of species and N is the total number of individuals in the sample. Although it attempts to mitigate for sampling effects, the Margalef index evaluates species richness and is very sensitive to sample size (Magurran, 2004). The species richness (total number of species in each sample) and the Margalef index were computed using either the absolute number of individuals or the density. The percentage variation was calculated by dividing the Margalef index obtained from the density matrix by the Margalef index obtained from the absolute number matrix (Gamito, 2010).

Pielou's evenness index, J' = H/ln S (Pielou, 1996)

Here, J′ is the similarity or evenness index, S is the total number of species, ln is the natural logarithm and H′ is the Shannon-Weaver index. Pielou's evenness index represents the probability that two individuals, picked independently and at random from a population, will belong to different species (DeJong, 1975).

### 2.5 Catch per unit effort (CPUE) and gear efficiency

The catch per unit effort (CPUE) of the angling gears were taken dependent on the weight of fish discovered amid an angling day (kg gear^−1^haul^−1^) for the various species consolidated and the gear efficiency (kg gear^−1^person^−1^hour^−1^) additionally assessed based on the weight of fish got and a number of individuals drew in with each fishing gear per hour.

### 2.6 Statistical analyses

Tabular technique was applied for processing the data by using simple statistical tools like averages and percentages. Hierarchical agglomerative clustering with group average linking and nonmetric multidimensional scaling (nMDS) were performed to investigate similarities among stations and months. The community succession at three stations during 12 months was summarized using the submodule of CLUSTER of Bray-Curtis similarities from species abundance. The multivariate Cluster and nMDS analyses were performed using the software PRIMER (Plymouth Routines Multivariate Ecological Research) v7.0.13 (Clarke and Gorley, 2015). The differences in CPUE, species composition and gear efficiency of the catch between months and fishing sites were analyzed, employing analysis of variance (ANOVA) techniques. Tukey's *post hoc* tests were used to compare the significant differences (p < 0.05) in the gear efficiencies of different sites and mean monthly variations of fish catch. All the data were analyzed by using Origin V9 and Minitab V17 software and the differences were significant at p-values of less than 5%.

## 3 Results

### 3.1 Fish biodiversity status

During the study period, a total of 62 fish and 2 prawn species under 12 orders and 24 families were recorded. Among these 64 species, 50 species were found in the catch assessment period, but according to the statement of fishermen and record from Upazila Fisheries Office another 14 species also found in the study area (Table 1). Cypriniformes order contributed highest (27%, 18 species) in which Cyprinidae family along contributed 25% (16 species) out of 24 families. According to the IUCN red list 2015 of Bangladesh, 2 species were critically endangered, 7 species were endangered and 6 species were found as vulnerable.

**Table 1:** Present status of fish diversity in the Gorai River.

Order	Family	English name	Scientific name	Present status	**IUCN status (BD)**	**IUCN status (Global)**
Beloniformes	Beloniidae	Freshwater gar fish	*Xenentodon cancila*	A	LC	NE
Clupeiformes	Clupeidae	Hilsa shad	*Tenualosa ilisha*	A	LC	LC
		Indian river shad	*Gudusia chapra*	A	VU	LC
		Ganges river sprat	*Corica soborna*	A	LC	LC
	Engraulidae	Gangetic hairfin anchovy	*Setipinna phasa*	VR	LC	LC
Channiformes	Channidae	Spotted snakehead∗	*Channa punctata*	A	LC	LC
		Asiatic snakehead∗	*C. orientalis*	R	LC	LC
		Snakehead murrel	*C. striatus*	LA	LC	LC
		Giant snakehead∗	*C. marulius*	R	EN	LC
Cypriniformes	Cobitidae	Guntea loach	*Lepidocephalichthys guntea*	A	LC	LC
		Necktie loach	*Botia dario*	R	EN	LC
	Cyprinidae	Indian major carp	*Labeo catla*	LA	LC	NE
		Indian major carp	*Labeo rohita*	LA	LC	LC
		Indian major carp	*Cirrhinus cirrhosus*	R	NT	VU
		Reba carp	*C. reba*	A	NT	LC
		Carplet/Morari	*Cabdio morar*	A	LC	LC
		Bata	*Labeo bata*	LA	LC	LC
		Black Rohu	*L. calbasu*	LA	LC	LC
		Fine scale razorbelly minnow∗	*Chela cachius*	R	VU	LC
		Large razorbelly minnow	*Salmostoma bacaila*	A	LC	LC
		Fine scale razorbelly minnow	*S. phulo*	A	NT	LC
		Mola Carplet	*Amblypharyngodon mola*	LA	LC	LC
		Flaying barb∗	*Esomus danrica*	LA	LC	LC
		Cotio	*Osteobrama cotio*	R	NT	LC
		Ticto barb	*Pethia ticto*	LA	VU	LC
		Spot fin swamp barb	*P. sophore*	A	LC	LC
		Olive barb∗	*Systomus sarana*	R	NT	LC
Decapoda	Palaemonidae	Monsoon river prawn	*Macrobrachium malcolmsonii*	A	LC	NE
		Monsoon river prawn	*M. lamarrei*	A	LC	NE
Mugiliformes	Mugillidae	Mullet	*Rhinomugil corsula*	A	LC	LC
Osteoglossiformes	Notopteriidae	Clown knife fish	*Chitala chitala*	VR	EN	NT
Perciformes	Ambassidae	Elongated glass perchlet	*Chanda nama*	LA	LC	LC
		Highfin glassy perchlet	*Parambasis lala*	A	LC	NE
		Indian glassy fish	*P. ranga*	A	LC	LC
		Climbing perch	*Anabas testudineus*	LA	LC	DD
	Badidae	*Badis* and Dwarf chameleon fish	*Badis badis*	R	NT	LC
	Gobiidae	Tank goby	*Glossogobius giuris*	A	LC	LC
	Osphronemidae	Honey gourami	*Trichogaster chuna*	LA	LC	LC
		Dwarf gourami	*Trichogaster lalia*	R	LC	LC
	Nandidae	Mud perch	*Nandus nandus*	R	NT	LC
	Sciaenidae	Pama croaker	*Otolithoides pama*	A	NE	NE
Siluriformes	Bagridae	Day's mystus	*Mystus bleekeri*	LA	LC	LC
		Tengara mystus	*M. tengara*	A	LC	LC
		Striped dwarf catfish	*M. vittatus*	A	LC	LC
		Long whiskered catfish	*Sperata aor*	LA	VU	LC
		Rita	*Rita rita*	VR	EN	LC
	Heteropneustidae	Stinging catfish	*Heteropneustes fossilis*	LA	LC	LC
	Pangasiidae	Yellow tail catfish∗	*Pangasius pangasius*	VR	EN	LC
	Schilbeidae	Batchwa vacha	*Eutropiichthys vacha*	A	LC	LC
		Murius vacha	*E. murius*	A	LC	LC
		Garua Bachcha	*Clupisoma garua*	A	EN	NE
		Gangetic ailia	*Ailia coila*	LA	LC	NT
		Indian potasi	*Pachyterus atherinoides*	R	LC	LC
		Silond catfish∗	*Silonia silondia*	VR	LC	LC
	Siluridae	Freshwater shark	*Wallago attu*	LA	VU	NT
		Pabo catfish	*Ompok pabo*	VR	CR	NT
	Sisoridae	Dwarf goonch	*Bagarius bagarius*	VR	CR	NT
		Indian gagata	*Gagata cenia*	A	LC	LC
Synbranchiformes	Mastacembelidae	Zig-zag eel	*Mastacembelus armatus*	A	EN	NE
		Barred spiny eel	*Macrognathus pancalus*	LA	LC	LC
		Lesser spiny eel∗	*M. aculeatus*	R	NT	NE
		Mud eel∗	*Monopterus cuchia*	R	VU	VU
Tetraodontiformes	Tetraodontidae	Ocellated pufferfish	*Leiodon cutcutia*	LA	LC	LC
Cyprinodontiformes	Aplochaeilidae	Blue panchax∗	*Aplocheilus panchax*	R	LC	LC

∗ Species not found during catch assessment but also reported from local fishermen and UFO. R: rare, VR: very rare A: available, LA: less available, EN: endangered, CR: critically endangered, VU: vulnerable, NE: not evaluated, NT: near threatened, LC: least concern, DD: data deficient, and EX: exotic species, BD: Bangladesh, IUCN status (IUCN 2015).

### 3.2 Abundance of ten most available fish species

**Figure 2:**
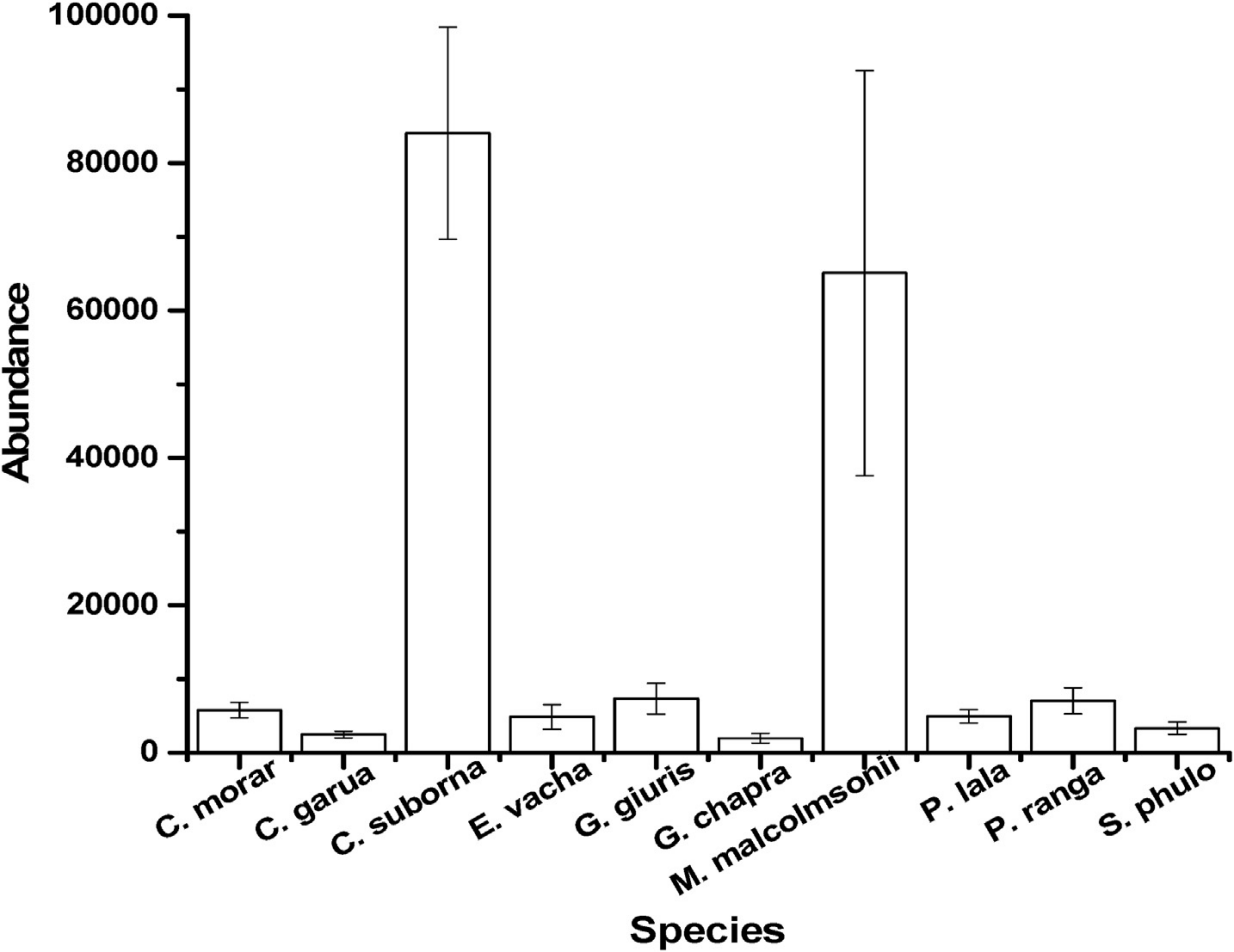
Abundance of 10 most available fish species found in the study area. Values are mean ± SD.

During the study period, a total of 10 most abundant fish species were identified among the 64 available fish species (Figure 2). Kachki (*Corica soborna*) ranked as the highest with the number of 84061 ± 14378 and followed by *Macrobrachium malcolmsonii* (65073 ± 27469), *Glossogobius giuris* (7317 ± 2108), *Parambassis ranga* (7038 ± 1783), *Cabdio morar* (5778 ± 1056), *Parambassis lala* (4930 ± 927), *Eutropiicthys vacha* (4855 ± 1668), *Salmostoma phulo* (3327 ± 797), *Clupisoma garua* (2489 ± 460) and *Gudusia chapra* (1976 ± 667). Though these species were found available in every month of the study period but the intensity of abundance varied with the different months and different sampling sites.

### 3.3 Diversity indices

Diversity was highest (H' = 2.245, 1-D = 0.84) in June and lowest in September (H' = 0.635, 1-D = 0.21); richness was highest (d = 22.035) in September and lowest in August (d = 7.033) and the values of evenness index (J′) was recorded highest (J' = 0.769) in August and lowest in September (J' = 0.239). The mean value of Shannon-Weaver diversity (H′), Simpson's index (1-D), Margalef's richness (d) and Pielou's evenness (J′) indices were recorded as, 1.478 ± 0.495, 0.57 ± 0.197, 15.115 ± 4.435 and 0.481 ± 0.152, respectively (Table 2).

**Table 2:** Number of calculated species, individuals, and values of Shannon-Weaver diversity, Simpson's index, Margalef's richness and Pielou's evenness indices in each sampling month.

Month	Number of species (S)	Diversity, H′	Simpson's, 1-D	Richness, d	Evenness, J′
January’18	21	1.283	0.54	16.371	0.415
February’18	23	1.116	0.41	19.716	0.356
March’18	30	1.619	0.66	17.914	0.476
April’18	31	1.575	0.62	19.052	0.459
May’18	28	1.639	0.69	16.477	0.492
June’18	32	2.245	0.84	13.811	0.648
July’18	22	1.928	0.72	10.894	0.624
August’18	16	2.133	0.82	7.033	0.769
September’18	15	0.635	0.21	22.035	0.235
October’18	16	1.011	0.37	14.842	0.365
November’18	16	1.621	0.61	9.252	0.585
December’18	14	0.930	0.35	13.983	0.352
Average	22	1.478 ± 0.495	0.57 ± 0.197	15.115 ± 4.435	0.481 ± 0.152

### 3.4 Cluster analysis

**Figure 3:**
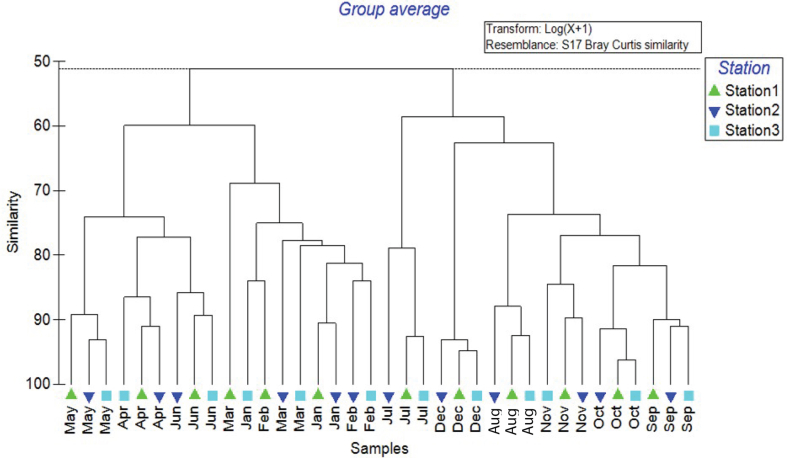
Dendrogram of clusters based on Bray-Curtis similarity matrix of different months and stations showing structural variability of the fish communities.

Cluster analysis revealed a clear structural variation in fish communities among the three stations in twelve months (Figure 3). At the similarity level of 58.7% separation, two major clusters were observed. The first cluster consists of January, February, March, April, May, June, and the second cluster consist of July, August, September, October, November, and December for station 1, station 2, and station 3.

### 3.5 Non-metric multidimensional scaling (nMDS)

**Figure 4:**
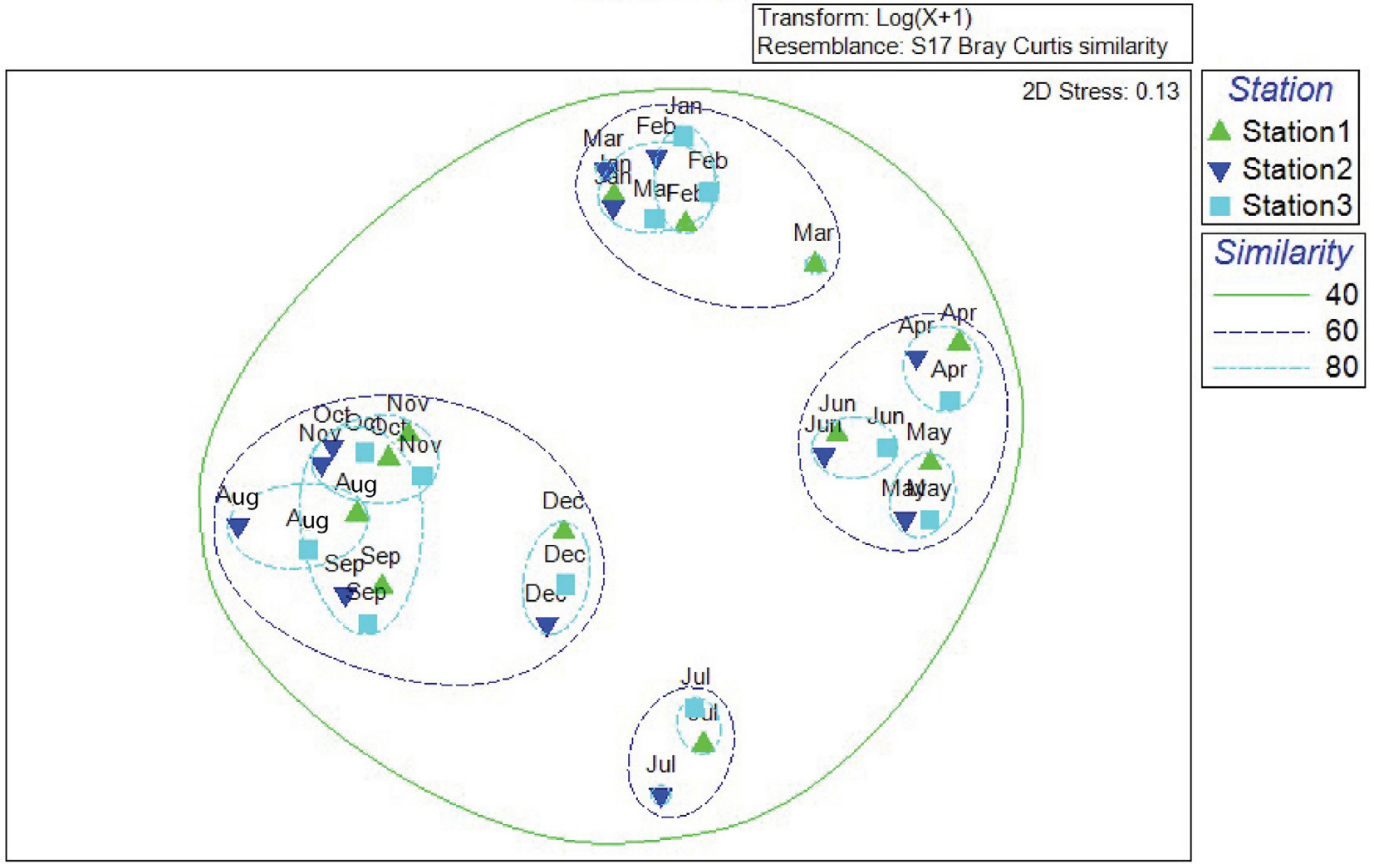
Dendrogram of the species distribution at three stations in twelve months using group average clustering based on Bray-Curtis similarities coordinating non-matric multidimensional scaling (nMDS) from square root transformed species abundance data of each species.

Non-metric Multidimensional Scaling (nMDS) analysis was performed to investigate similarities among fish abundance. nMDS showed 40% similarity for all months, while 60% similarity showed four marked separations in the fish abundance in different months where only July formed a single cluster with three stations. However, at 80% similarity, overlay clusters were observed for August, September, October, and November with all stations and, another overlay cluster was found in January, February, and March (except station 1 in March). The individual and separate clusters were observed for April, May, June, July, and December with three stations respectively (Figure 4).

### 3.6 Fishing crafts and gears

About 176 fishing boats were recorded from the study area used by the fisherman for fishing purpose. Among them 36 were mechanized, 111 were non-mechanized and 29 were small fishing craft (locally known as talo dingi). The range of diesel engines used for operating the mechanized fishing boat varied from 2 to 40 HP. On the other hand, non-mechanized and small fishing craft operated manually by one or more fishermen. The range of the length of mechanized fishing craft varied from 30 to 40 ft. and non-mechanized fishing boats was 20–25 ft. long. Most of the fishermen used wooden boat for fishing in the Gorai River. Amidst the time of study, 10 unique gears under 4 categories were found to operate in the Gorai River. A large portion of the fishing gears was utilized in the pre-monsoon and after the monsoon and a portion of all year equips likewise recorded from the sampling area (Table 3).

**Table 3:** Types of fishing gears and period of operation in the study area.

Category	Type of gear	Name of gear	Person engaged	Mesh size (cm)	Target species	Period of operation
Fish net	Seine net	Ber jal	12–14	0.25–1	All	December–June
		Jangle jal	10–12	Fine	All	November–May
	Gill net	Current jal	1–2	2.5–10	All	Year round
	Lift net	Dharma jal	1–2	0–0.5	All	August–November
	Drift gill net	Chandi jal	3–4	3–6	Ilish	August–October
	Drag net/push net	Thala jal	1	0–0.25	All	Year round
	Cast net	Jhaki jal	1–2	0.5–1	All	Year round
Hook & line	Hook & line	Chip borshi	1–2	-	Carnivorous	August–October
Fish trap	Fish Trap	Dhoar	1–2	-	Punti, Baim	April–August
Wounding gear	Wounding gear	Koch/juti	1	-	Large fish	Year round

### 3.7 Catch composition of different fishing gears

**Figure 5:**
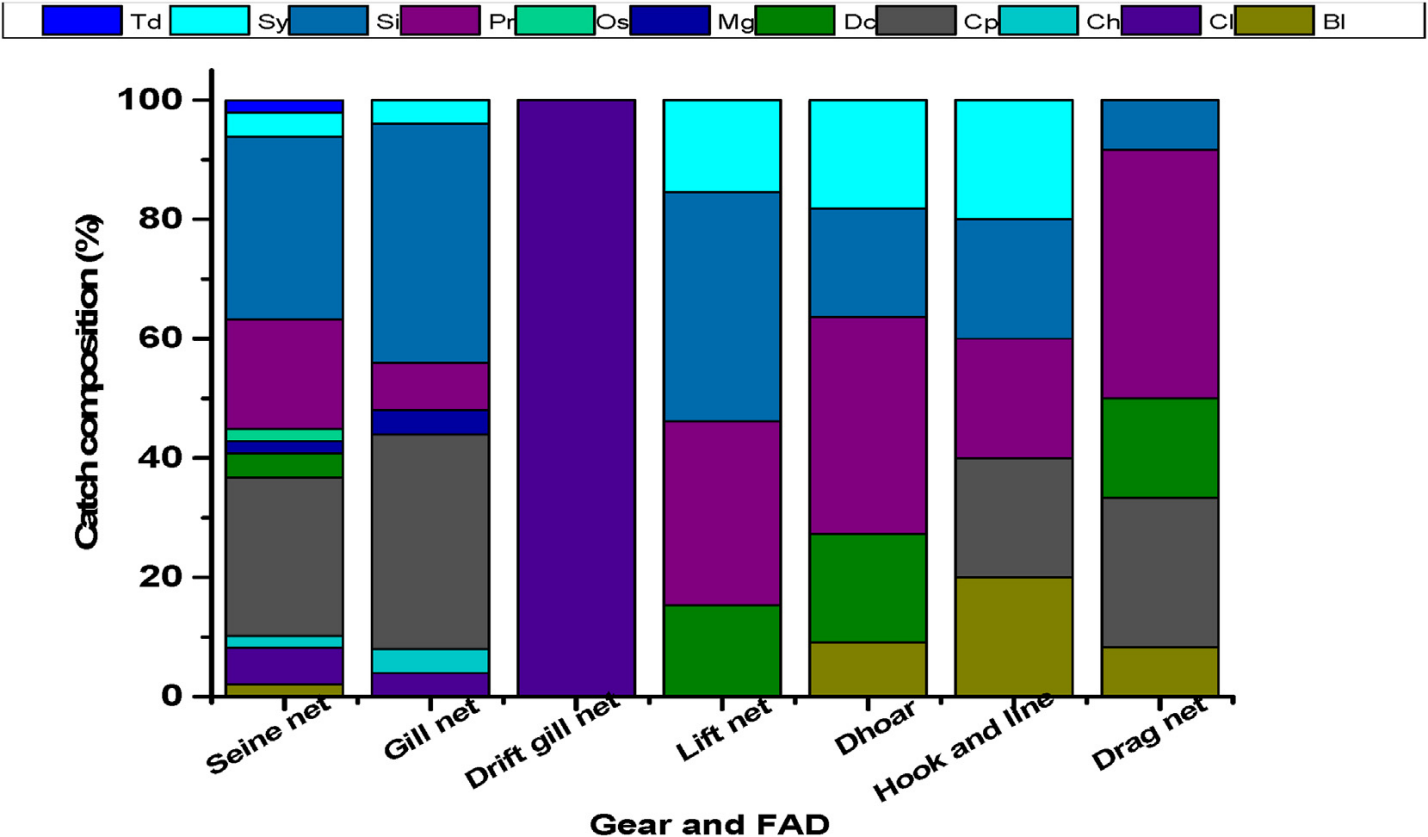
Percentage compositions of different categories of fish harvested with different fishing gear. FAD: Fish aggregating devices, Td: Tetraodontiformes, Sy: Synbranchiformes, Sl: Siluriformes, Pr: Perciformes, Os: Osteoglossiformes, Mg: Mugiliformes, Dc: Decapoda, Cp: Cypriniformes, Ch: Channiformes, Cl: Clupeiformes, Bl: Beloniformes.

Maximum 50 fish species caught by using seine net where maximum 30.60% fish species were belonging to the Siluriformes order and minimum 2.04% belonging to the Tetraodontiformes order. But in the case of Drift gill net, there were only one species (*Tenualosa ilisha*) caught by the gear belonging to the Clupeiformes order which contributes 100% of the total fish caught by the drift gill net in the study area (Figure 5). Each different fishing gear can catch a large variety of species exist in most fishing grounds in the Gorai River.

### 3.8 CPUE and gear efficiency

**Figure 6:**
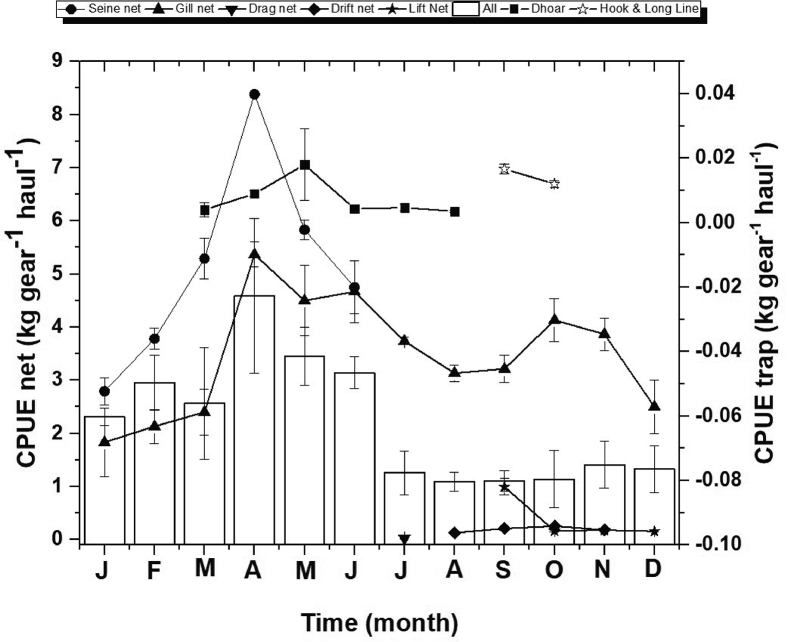
Monthly variations in CPUE of different fishing gears employed in the Gorai River.

During the survey period the highest CPUE (kg gear^−1^ haul^−1^) was recorded from seine net 5.2 ± 1.72 kg and lowest CPUE showed by hook and long lines 0.0135 ± 0.0015 kg in the study area. The maximum average CPUE 4.59 ± 3.46 kg of all gears and minimum average CPUE 1.09 ± 1.18 kg of all gears was recorded during April and August, respectively (Figure 6).

**Figure 7:**
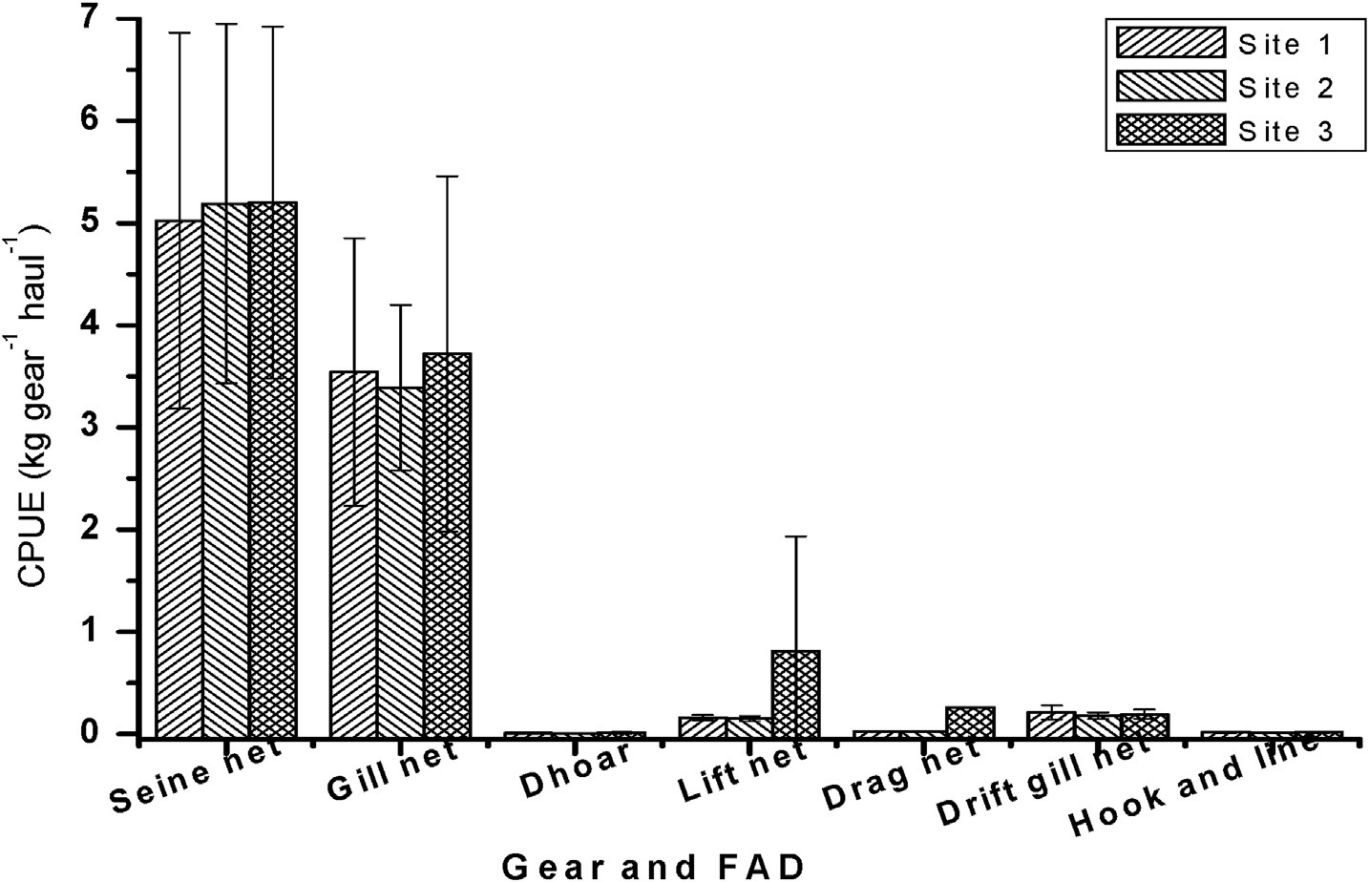
Variation of CPUE in three different sampling sites. Values are mean ± SD.

The CPUE of the 7 most used frequently available fishing gears in three different sites of the Gorai River are clarifying in Figure 7. Highest CPUE was recorded from seine net 5.2 ± 1.72 kg in site-3 and lowest 0.0135 ± 0.0015 kg from hook and long line in site-2.

**Figure 8:**
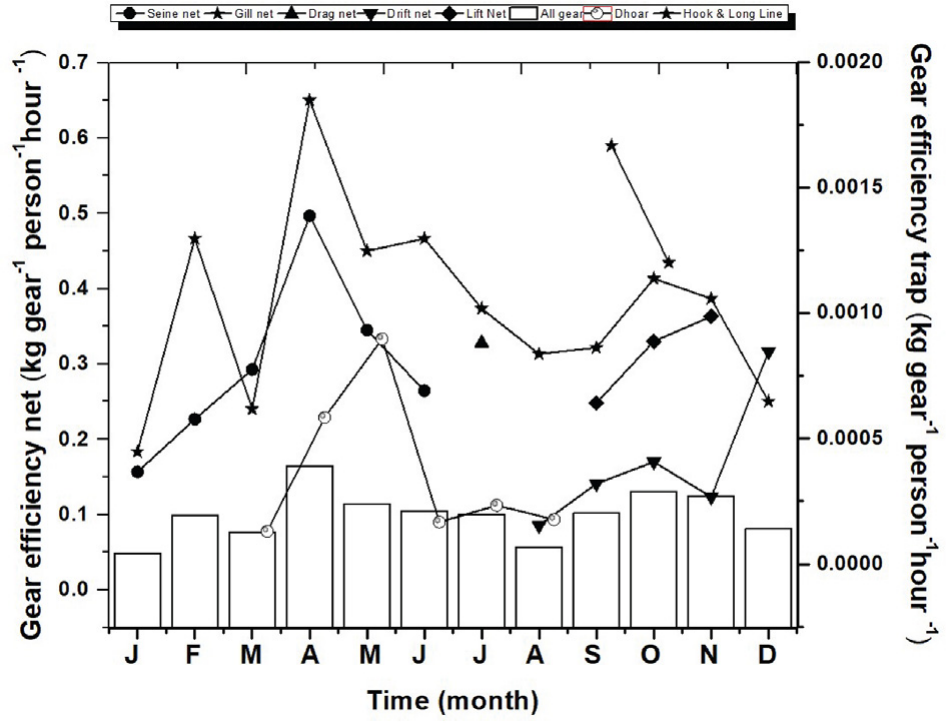
Monthly variations of gear efficiency of different gears frequently used in the Gorai River.

The monthly variations in gear efficiency (kg gear^−1^person^−1^hour^**−1**^) of frequently used gears and trap in three different sites of the Gorai River showed in Figure 8. The maximum level of gear efficiency (0.65 kg gear^−1^person^−1^hour^**−1**^) was recorded from the gill net during April and lowest 0.000897 kg was recorded during May. The maximum average gear efficiency (0.35 kg) of all gear was recorded during February and minimum average gear efficiency (0.08 kg) of all gear was recorded on March and October, respectively.

**Figure 9:**
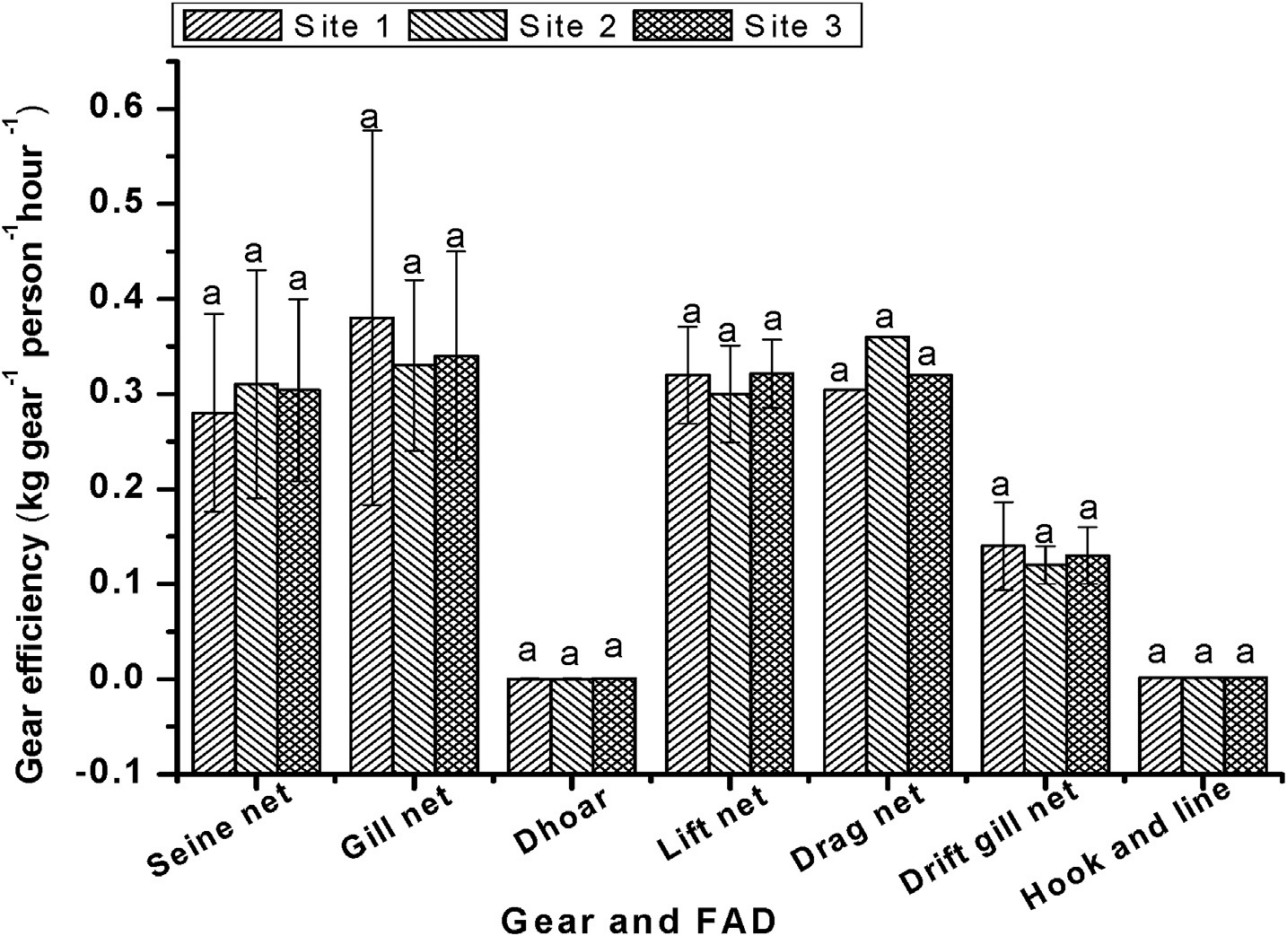
Variation of gear efficiency in three different sampling sites. Values are mean ± SD.

The gear efficiency (kg gear^−1^ person^−1^ hour^**−1**^) of the 7 most used frequently available fishing gears in three different sites of the Gorai River are displayed in Figure 9. The average gear efficiency of dhoar, and hook and line were significantly lower than others (p < 0.05). However, gear efficiency of a specific gear in three different sites were not significantly different (p > 0.05). A fisherman used 150 to 200 or more dhoar and 400–500 or more hook to catch fish at a time. Though a single dhoar or hook can catch a small amount of fish but the total catch of these large number of dhoar or hook become more or less equal to a gill net or a lift net. Mainly carnivorous fish species were targeted to catch through dhoar and hook, and their economic values were not so different from other species.

### 3.9 Total fish catch in the study area

**Figure 10:**
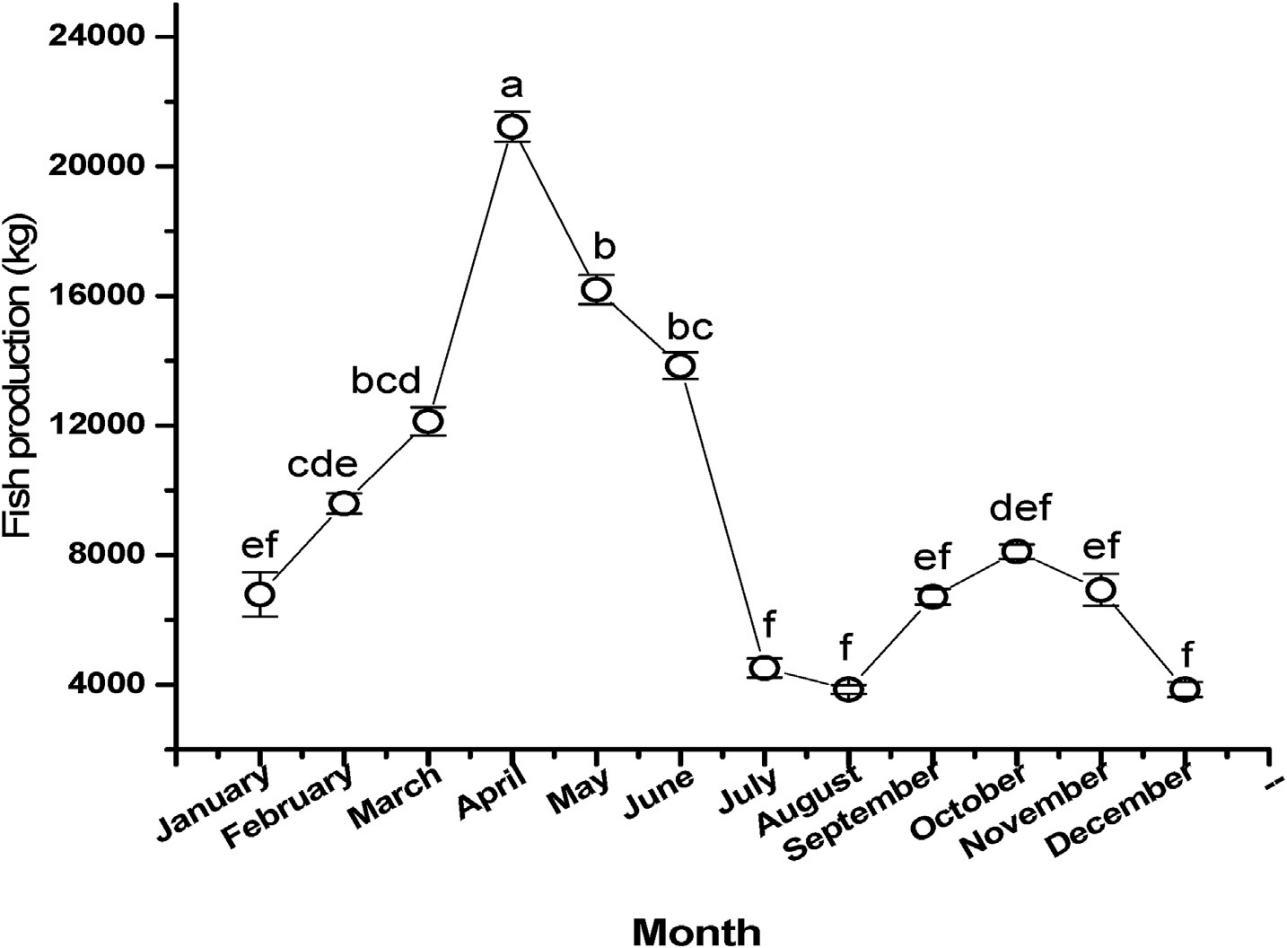
Month wise variation in fish catch of the Gorai River. Values are mean ± SD.

The month-wise fish catch of three sampling sites of the Gorai River was recorded by catch assessment method (Figure 10). The highest fish catch 21228 ± 464.38 kg was recorded from April and lowest 3855 ± 138.21 kg during August. However, the mean monthly fish catch in April was significantly higher (P < 0.05) from other months.

### 3.10 Decline causes of fish diversity in the Gorai River

The threats to fisheries biodiversity can be described under some interacting categories such as Change of river course, overexploitation of fisheries resources, destruction of habitat, and water quality deterioration by pollution. Due to the increasing population, overexploitation, use of illegal fishing gear and fishing pressure is increasing day by day. The river course was changed because of establishing dam, bridges across the river. As indicated by the respondents the interacting and combined influences of some natural and man-made causes under these major threat categories have resulted in a reduction of fisheries biodiversity of the Gorai River (Table 4).

**Table 4:** Causes of loss of fish diversity.

SL No.	Threats to fish diversity	No. of Respondent	Percentage of Respondents
1.	Siltation and sedimentation, decreasing the water depth of the river	78	98%
2.	High fishing pressure	70	88%
3.	Catching of brood fish, fry and fingerling through seine net	67	84%
4.	Use of illegal fishing gear like current jal	61	76%
5.	Practicing illegal and destructive fishing methods like poison fishing by gas tablet (Rotenone)	58	73%
6.	Increasing fishing pressure and fishing during breeding season	46	58%
7.	Construction of different types of flood control, development and communication infrastructures like bridge, dams, embankments, etc.	45	56%
8.	Creation of barrier and making obstacle in natural migratory route of fishes	41	51%
9.	Low water velocity (water current)	40	50%
10	Poor implementations of fishing rules and regulations	38	48%
11.	Use of insecticides and pesticides in agricultural crop land	36	45%
12.	Use of chemical fertilizers like urea, TSP, MoP etc.	35	44%
13.	To make agricultural crop land by filling the river side	33	41%
14.	Drought in summer season	30	38%
15.	Use of river water for irrigation purposes	16	20%

## 4 Discussions

### 4.1 Present status of fish biodiversity

In this study a total of 62 fish and 2 prawn species under 12 orders and 24 families were recorded. Among these Cypriniformes order contributed a maximum of 27% (18 fish species) and the Cyprinidae family contributed a maximum of 25% (16 fish species). Hanif et al. (2016) identified Cypriniformes as dominant order with 12 species in the Gorai River and Sultana et al. (2017) also found Cypriniformes (32.38%) was the most dominant order and Cyprinidae was the most dominant family contributing 20 species in wetlands of Chhatak, Bangladesh. Similar findings of Cyprinidae family were also reported from many other rivers of Bangladesh. In this study area, Cypriniformes order was the most members compared to the other orders because of the ideal environmental conditions and river bottom that this family prefers (Hanif et al., 2016).

In this study, 15 fish species were recorded as threatened. Hanif et al. (2016) recorded 16 species as threatened at the river Gorai and categorized as 7 vulnerable, 7 endangered and 2 species were critically endangered. These findings are more or less similar to the present study. But Rubel et al. (2016) found 30 species in the Lohalia River of Bangladesh among which 60% recorded as threatened and categorized as vulnerable (37%), endangered (17%) and critically endangered (6%). These findings are different from the present study due to differences in geographical location.

Hossain et al. (2009) found jat punti (*Puntius sophore*), tit punti (*Pethia ticto*) followed by chanda (*Chanda nama* and *Parambasis ranga*), chapila (*Gudusia chapra*) and tengra (*Mystus vittatus*) as the most dominant species in the Chalan beel of Bangladesh. Galib et al. (2013) recorded jat punti (*Puntius sophore*) as the most dominated species in the Halti beel of Bangladesh. Kamrujjaman and Nabi (2015) documented kalo bujuri (*Mystus tengara*), and jat punti (*Puntius sophore*) as the most abundant fish species from the Bangshi river of Bangladesh. These results are different from the present study due to the difference in geographical location of these water bodies, survey periods, choice of fishing gear, etc.

### 4.2 Diversity indices

Shannon-Weaver variety (H′) index considers each the number of species and the distribution of folks amongst species of Gorai River. During the study period, the highest value of H′ was found as 2.245 in June and the lowest value was found as 0.635 in September. The average value of the index was recorded as 1.478 (Table 2). In each case of the highest Shannon-Weaver, diversity index is involved with high individuals and the lowest diversity involved with a low number of individuals. Islam and Yasmin (2018) recorded diversity indices (H′) 0.122 to 0.634 on Dhaleshwari River of Bangladesh, Jewel et al. (2018) recorded overall values of the diversity index (H′) in the Atrai River of Bangladesh were 3.12, Rahman et al. (2015) carried out a study on the Talma River of Bangladesh and the average values of diversity (H′) were recorded 1.42. So, present study is supported by these findings which are slightly different from the present findings because of different geographical locations, survey periods, different fishing methods and choice of fishing gear. But, Biligrami (1988) recommended better condition of water body for fish diversity when H′ index ranged from 3.0-4.5. According to this recommendation, Gorai River is strongly degraded which led to decline the fish diversity. Simpson's dominance index gave the probability of any two individuals drawn at random sampling from an infinitely large community belonging to different species. The Simpson index is therefore expressed as 1-D. It's a species diversity index derived by Simpson in 1949. Simpson's index is heavily weighted towards the most abundant species in the sample while being less sensitive to species richness (Islam and Yasmin, 2018). In the present study, the highest Simpson Dominance index (1-D) value 0.84 was observed in June and the lowest 0.21 in September with a mean value of 0.57 ± 0.197 (Table 2). Islam and Yasmin (2018) recorded Simpson's index 0.325 to 0.893 in Dhaleshwari River, Bangladesh; which was higher than the present findings.

Margalef's richness is the simplest measure of biodiversity and is simply a count of the number of different species in a given area. This measure is strongly dependent on sampling size and effort (Siddique et al., 2016). In this study, the lowest and highest Margalef's richness index value was observed as 7.033 in August and 19.716 in February (Table 2), respectively. Most fish species started breeding from June when the monsoon start in Bangladesh which might be the purpose in the back of the lowest and very best richness value during August and February. As a result, numbers of new individuals joined the fish shares increased the species richness in winter (Siddique et al., 2016). Islam and Yasmin (2018) recorded richness index (d) 4.793 to 7.438 on Dhaleshwari River, Galib et al. (2013) calculated fish species richness value in the Choto Jamuna River of Bangladesh and found values varied from 6.973 in June to 8.932 in November, Jewel et al. (2018) recorded overall values of richness index (d) in the Atrai River was 5.87. Rahman et al. (2015) carried out a study on the Talma River and the average values of richness (d) index was recorded 6.64. The Margalef's index may deviate from actual diversity value to some extent because it does not confound the evenness and species richness value properly and it is depending on sample size (Nair et al., 1988). This might be occurred as a result of reduced water depth due to lack of rainfall, which disturbed fishermen to operate their fishing gears more effectively (Iqbal et al., 2015). Besides, ecological conditions also affected the distribution of the fish species (Siddique et al., 2016). Construction of several bridges on the river, heavy river erosion in the monsoon and construction of unruly earthen dam during lean period for fishing are the main causes for ecological degradation.

Pielou's evenness index (J′) measures the evenness in which individuals is divided among the taxa present (Siddique et al., 2016). During the study period, the recorded highest evenness (J′) value was found as 0.763 (September) and the lowest as 0.235 (September) whereas the average value was recorded as 0.481 in the sampling area of Gorai river (Table 2). Therefore, the species equitability index among the sampling area and in the different months reveals that the distribution of fish population of Gorai River is more or less equally distributed. The values are also close to the findings of Islam & Yasmin (2018); they recorded evenness index (J′) 0.117 to 0.588 in Dhaleshwari River, Jewel et al. (2018) recorded overall values of the evenness index (J′) in the Atrai River was 0.66, Rahman et al. (2015) carried out a study on the Talma River and the average values of Evenness (J′) index was recorded as 0.86. These findings are different from the present findings because of different geographical locations of the study. Murugan and Prabaharan (2012) found highest evenness value 0.99 at late monsoon indicating evenly distributed and rich fauna in the monsoon and post-monsoon.

### 4.3 Cluster analysis and non-metric multidimensional scaling (nMDS)

The cluster analysis showed distinct separation among the three sampling stations in twelve months. At the similarity of 58.7%, two major groups were attained. Nasren et al. (2021) found two cluster groups at 72.9% similarity in the Ratargul swamp forest where group A comprises the fish species of January, February, and March month and group B contains fish species of April to December month. Hossain et al. (2012) found two different clusters of fish species at the similarity of 32% in the Meghna River of Bangladesh. On the other hand, the Multidimensional Non-metric scaling (nMDS) showed an overall 40% similarity among the three stations in twelve months. Shamsuzzaman et al. (2016) found 20% similarities in all seasons in Karnafully river and Rashed-Un-Nabi et al. (2011) found 65% similarity for finfish and shellfish in all seasons in Bakkhali river estuary. Their findings are dissimilar from the present findings because of the different geographical locations, different survey periods, and sampling error variation.

### 4.4 Fishing gear, gear efficiency and fish catch

Rubel et al. (2016) recorded 11 types of fishing net under 5 main categories in the Lohalia River of Bangladesh of which ber jal under the group seine net, current jal, chandi jal under the group gill net was responsible for large scale catch. Ali et al. (2015) has identified eight major types of fishing gears using in the Ramnabad River. Sultana et al. (2016) recorded 18 types of fishing gear and 3 types of traps in the Payra River, Bangladesh. The previously documented studies on fishing gears are different from the present study. Because the choice of fishing gears by the fishermen depends on many factors like types of fish species available in the river, the physical condition of the river such as the presence of currents, bottom conditions, and types of aquatic vegetation present in the river. Some fishermen use illegal fishing gear like fine-meshed seine net (locally called Jangle jal), gill net (current jal), and illegal fishing method like poison fishing in the Gorai River. Which are adversely affects the fish biodiversity and causes the extinction of many fish species from the river. In addition to fishing gears, fishers of Bangladesh also use other methods such as fishing in ditches and the draining of canals, sections of small river channels, and ponds (Craig et al., 2004).

Saberin et al. (2018) recorded a total of 19 types of fishing gear in the Old Brahmaputra River from April 2011 to March 2012. Among which Seine net showed the highest CPUE of 5.56 kg gear^−1^day^−1^ with fishing effort 0.0224 gear^−1^haul^−1^day^−1^ followed by push net and lift net. Ahmed and Hambery (2005) recorded the CPUE ranged between 2.91 and 30.86 kg gear^−1^day^−1^. Sayeed et al. (2014) recorded a total of 34 different types of fishing gears operated in Chalan beel in which seine nets were the dominant gear followed by gill nets and set bag nets. These previously documented study on CPUE is different from the present study due to the difference of fishing places, the net sizes, the number of hooks, lures, and baits, etc.

In the present study it was found that fish catch in the Gorai River was higher in the pre-monsoon period and lower during the monsoon and winter period. Hossain et al. (2009) recorded a total of 12,217 tons of annual fish production in the Chalan beel during 2005–2006 which was half of the production observed in 1982. Ahmed and Hambery (2005) found that the production and richness of fish fauna are bound to the inundating pattern in the monsoon period. These production patterns are similar to the present study. The availability of the fish species was comparatively higher in the pre-monsoon season due to the optimum level of water and temperature but during the post-monsoon water current and water level increases which made the fishing activities very difficult. That's why the total fish catch becomes low during August.

### 4.5 Threats on fish biodiversity

Anthropogenic and natural hazards increasing day by day and squeeze the fish species distribution across the country (Sarkar et al., 2008) and subsequently, many fish species are documented as endangered in Bangladesh (IUCN Bangladesh, 2015). A large number of indigenous fish species and some anadromous fish species use the Gorai River as a major feeding, breeding ground, and migratory route (Hanif et al., 2016). But in recent years the riverine ecosystem in Bangladesh has changed considerably due to pollution, human intervention, and global warming which have destroyed the riverine ecosystem (Alam et al., 2017; Islam et al., 2015, 2017). Habitat destruction, reduced water flow, indiscriminate fishing of fry, and fingerling are also considered as significant factors for declining fish species diversity in Bangladesh (Sultana et al., 2019; Pandit et al., 2015; Rahman et al., 2012). These researches found similar declining causes that represent the declining trends of fish diversity in the study area which warning the gradual declination of fish diversity of Bangladesh.

## 5 Conclusion and recommendations

Gorai River is a moderate productive water body with diminishing fish species of decent variety. Species selectivity of various gear contrasted significantly. Gill nets and fine coincided seine nets were discovered more destructive than those of different gears. These sorts of unlawful fishing rehearse were across the board and asset poor fishers proceeded these for their employee as they couldn't discover other elective works amid the periods. This research is a primer endeavor to think about fish diversity index, gear efficiency, CPUE and catch composition of various fishing gears and reasons for decrease of fish fauna in Gorai River. Subsequently, fishing ought to be prohibited amid breeding seasons by NGOs and government just as fisheries look into foundation. Fishing gears ought to be developed relying upon target fish species. Fishing nets (seine net and gill net) with large mesh size would be a potential helpful gear for conservation of fish species. The followings are recommended for policy making, implementation, and conservation of fish biodiversity in the Gorai River:

➢ Banning of indiscriminate killing of brood fish and fry/fingerlings.
➢ Banning or controlling destructive fishing gears like current jal and destructive fishing methods like fishing by poisoning.
➢ Identification of the fish breeding and nursery grounds and its protection.
➢ Identification of fish migration and fish breeding period of different indigenous fish species.
➢ Establish a sufficient number of fish sanctuaries and ensure proper maintenance of them.
➢ Dredging river bed for continuous river water flow to facilitate fish migration.
➢ Minimizing the uses of harmful insecticides and pesticides in agricultural crops.
➢ Providing alternative income opportunities to the poor fisherman during the banned fishing period.
➢ National strategies are formulated for policymaking, monitoring, and implementation of the Gorai River.
➢ Overall public awareness should be expanded through training program to restore the habitat of these valuable fish species from close extinction.

## Declarations

### Author contribution statement

Kishor Kumar Tikadar: Conceived and designed the experiments; Performed the experiments; Contributed reagents, materials, analysis tools or data; Wrote the paper.

Mrityunjoy Kunda: Conceived and designed the experiments; Analyzed and interpreted the data; Contributed reagents, materials, analysis tools or data.

Sabuj Kanti Mazumder: Analyzed and interpreted the data.

### Funding statement

This work was supported by the Ministry of National Science and Technology, Peoples Republic of Bangladesh (2017–2018 FY).

### Data availability statement

Data included in article/supplementary material/referenced in article.

### Declaration of interests statement

The authors declare no conflict of interest.

### Additional information

No additional information is available for this paper.
